# Integration of specialized residential interventions with community-based services for autistic adults with severe challenging behaviors: preliminary evidence from the Piedmont model

**DOI:** 10.3389/fpsyt.2026.1925564

**Published:** 2026-08-05

**Authors:** Roberto Keller, Martina Giacchero, Francesca Capiotto, Martina Masoero, Stefano Corna, Giusi Figliano, Marilia Barbosa De Matos, Donato Liloia

**Affiliations:** 1 Adult Autism Centre, Mental Health Department, Local Health Unit ASL Città di Torino, Turin, Italy; 2 Istituti Clinici Scientifici Maugeri IRCCS, Institute of Veruno, Gattico-Veruno, Italy; 3 Department of Psychology, University of Turin, Turin, Italy

**Keywords:** adulthood, applied behavior analysis, autism spectrum disorder, challenging behaviors, inpatient care, specialized care pathway

## Abstract

**Background:**

Severe challenging behaviors in autistic adults are difficult to manage in generic psychiatric settings and impose a heavy burden on individuals, families, and services. The Piedmont Region (Italy) established a specialized third-level inpatient unit for these presentations, embedded within an integrated, three-tier regional network. This study reports preliminary effectiveness data from this model.

**Materials and methods:**

In a prospective, pre-post design, 10 autistic adult males admitted for severe challenging behaviors received an intensive, individualized psychoeducational program based on Applied Behavior Analysis during a time-limited specialized admission. Psychopathology (Brief Psychiatric Rating Scale, BPRS), challenging behaviors (Aberrant Behavior Checklist, ABC), global clinical severity (Clinical Global Impression, CGI), and social support (Social and Affective Functional Support Scale, SSAF) were assessed before admission (T0) and at discharge (T1). Change was examined with paired-samples t-tests and standardized effect sizes; given the sample size and number of comparisons, analyses were exploratory.

**Results:**

Improvement was selective rather than uniform. On the BPRS, significant reductions emerged across several dimensions, concentrated in a coherent behavioral-disorganization and agitation cluster (i.e., motor tension, distractibility, bizarre behavior, conceptual disorganization, and anxiety) with large effect sizes. The ABC showed significant reductions in lethargy/social withdrawal and hyperactivity, whereas stereotypic behavior, irritability, and inappropriate speech did not change significantly. CGI and SSAF showed non-significant trends toward improvement. Trait-like, affective, and global dimensions appeared least responsive to the admission.

**Conclusion:**

A time-limited, specialized inpatient program delivered within an integrated regional network was associated with improvement concentrated in observable, modifiable behavioral dimensions. These preliminary findings support the development of specialized, coordinated care pathways for severe challenging behaviors in autistic adults and warrant controlled studies with larger, sex-diverse samples and post-discharge follow-up.

## Introduction

Autism spectrum disorder (ASD) is a lifelong neurodevelopmental condition defined by persistent differences in social communication and reciprocal interaction, restricted and repetitive patterns of behavior and interests, and atypical sensory processing (1). Because these features vary widely in expression and intensity, the clinical needs of autistic people differ markedly across individuals and across the lifespan (2–6). For a subset of autistic people, most often those with co-occurring intellectual disability and limited spoken language, adolescence and adulthood bring behaviors that are severe, persistent, and difficult to contain: aggression, self-injury, and prolonged agitation, commonly grouped under the term *challenging behaviors* (7–9). These are among the most consequential problems in adult autism care. They erode the autistic person’s participation and autonomy, shape the quality of life of entire families and, when they escalate, precipitate emergency presentations, psychiatric admission, and the use of coercive measures (10). Their assessment and management are correspondingly demanding, and the stakes are high for the individual, the family, and the service system (8, 11).

Current guidance, including that of the Italian National Institute of Health (Istituto Superiore di Sanità 2025), converges on the view that no single intervention is appropriate for all autistic presentations; rather, care should be personalized, intensive, and delivered by a multidisciplinary team (12). Therefore, addressing severe challenging behavior requires a structured, sequential process. Organic and medical contributors must first be excluded, an especially important step in non-speaking individuals, in whom somatic pain, gastrointestinal disturbance, or epilepsy may underlie an apparent behavioral crisis (13). Environmental and contextual triggers, such as sensory load or disruption of routine, must then be identified. Beyond these precipitants, chronic challenging behaviors are frequently learned responses maintained by their consequences: they serve a function for the individual while simultaneously interfering with adaptive functioning, social participation, and overall quality of life (8, 14, 15). Functional behavior assessment seeks to identify the antecedents and consequences that maintain a behavior, providing the empirical basis for individualized intervention (16–18).

Applied Behavior Analysis (ABA) operationalizes these principles through the systematic use of reinforcement, extinction, shaping, and related procedures to increase adaptive behavior and reduce behavior that is harmful or maladaptive (19, 20). Meta-analytic evidence supports the efficacy of intensive ABA intervention for communication, adaptive, and cognitive skills in autistic children, particularly when delivered early and at high intensity (21–25). However, the evidence base for adults is considerably weaker. A systematic review identified only a small number of eligible studies, most of which used single-case or non-randomized designs; the reported effects were promising but heterogeneous and supported by low-certainty evidence (26). The extension of behavioral approaches to autistic adults also warrants caution on conceptual and ethical grounds, given ongoing debate about the social validity of behavior-reduction goals and the priority that autistic people and their families place on well-being, autonomy, and the least restrictive effective intervention (27–30). Taken together, these considerations argue for delivering behavioral intervention within a comprehensive, medically informed, and person-centered framework rather than as an isolated technique, and they underscore the need for empirical evaluation of such integrated models in the adult population.

The setting in which intensive intervention is delivered is itself consequential. General psychiatric inpatient wards may expose autistic people in crisis to high sensory and social load, including noise, bright lighting, alarms, crowding, disrupted routines, and unpredictability; these conditions conflict with autism-informed principles that emphasize structure, predictability, low arousal, individual sensory adaptations, and co-regulation, and may contribute to dysregulation, overwhelm, and escalation of the behaviors that admission is intended to contain (31, 32). Accumulating evidence indicates that specialized inpatient environments yield better outcomes than general units for this population (33). Resistant behavioral crises associated with autism and intellectual disability can stabilize within dedicated, interdisciplinary neurobehavioral units (13, 34, 35). Nonetheless, such services remain scarce, are concentrated in childhood and adolescence, and are seldom embedded within the wider system of community care; the limited capacity of autism-specialized inpatient provision for adults is a recognized gap internationally (36). Importantly, few published and outcome-evaluated models have described specialized residential or supported-living interventions for autistic adults with severe challenging behaviors that are explicitly embedded within wider community health and social-care pathways (37–41). This gap appears particularly pronounced in Italy, where adult autism services and residential initiatives exist. However, the available evidence remains largely service-descriptive rather than grounded in systematically evaluated, adult-specific residential models (42, 43).

The Piedmont Region in north-western Italy has recently developed such a model. Its care system is organized in a structured network across three levels: first-level autism outpatient clinics and community mental health centers located within each local health authority (Azienda Sanitaria Locale, ASL); two regional second-level autism centers, one for children and one for adults, providing specialist multidisciplinary consultation to local services (12, 44); and, most recently, a dedicated third-level inpatient facility for autistic individuals with severe challenging behaviors, structurally independent of standard acute psychiatric wards. Located at IRCCS Maugeri, this unit provides diagnostic assessment, ABA-based functional analysis, medical and pharmacological re-evaluation, and stabilization of complex presentations through an intensive multi-month protocol grounded in national guidelines (Istituto Superiore di Sanità 2025; Società Italiana di Neuropsichiatria dell’Infanzia e dell’Adolescenza 2005). Crucially, the unit is not conceived as a site for acute crisis containment alone; it operates in continuous coordination with the referring territorial teams before admission and with structured post-discharge projects afterwards, forming an integrated “*Piedmont model*” of care. This configuration anticipates the model that the Istituto Superiore di Sanità is now promoting nationally through EDECO (Equipe Dedicate alle Emergenze Comportamentali; Dedicated Teams for Behavioral Emergencies), a program that structures the response to challenging behavior in ASD across three levels of increasing specialization and complexity. First-level teams, widely distributed across the territory, provide first-line assessment and intervention for outpatients with ASD and behavioral difficulties; second-level teams—comprising psychiatrists and psychologists trained in cognitive-behavioral treatments specific to challenging behavior in ASD—deliver more specialized intervention and support the first-level teams; and third-level teams provide inpatient care for individuals with severe challenging behavior who require admission to a ward dedicated to ASD and kept distinct from general psychiatric settings. The Piedmont system described above can thus be regarded as an early, fully operational instance of this three-tier logic.

Against this background, the present study reports preliminary, real-world data on the intensive protocol delivered at IRCCS Maugeri, using a within-subject pre-post design. Standardized instruments were administered before admission and after discharge by the referring ASL services to capture several domains, including challenging behaviors and their functional characteristics, psychiatric symptom severity, global clinical status, and clinical change. Given its naturalistic design and small sample size, the present study should be regarded as an exploratory evaluation of the preliminary effectiveness of the integrated inpatient treatment model, rather than as a confirmatory trial. Consistent with the rationale for individualized, ABA-informed intervention embedded within comprehensive medical care, we anticipated reductions in challenging behaviors and in associated psychopathological symptoms, while expecting that broader, more structural indices of autism severity and support need would be less likely to change over the course of a relatively short admission.

## Materials and methods

### Structured intervention and unit

The third-level service at Istituto di Ricovero e Cura a Carattere Scientifico (IRCCS) Maugeri (https://icsmaugeri.it/) is organized as a specialized unit dedicated to managing severe, challenging behaviors in adolescents (aged 14–18 years) and adults diagnosed with ASD, operating through an integrated psychoeducational and clinical approach. The multidisciplinary team includes psychiatrists, child neurologists, psychologists, behavior analysts, professional educators, nurses, and healthcare support staff, ensuring integrated, comprehensive, and coordinated care. Referral and subsequent admission to IRCCS Maugeri are determined by the regional autism units for children or adults within the territorial ASL. Inpatient admission is considered the most appropriate therapeutic setting for individuals whose challenging behaviors require intensive management within a structured and highly specialized environment. Referrals are therefore made by the ASL services to Maugeri, which, following an initial meeting with the patient and family, admits the individual to the unit and develops an individualized intervention plan. Within this three-tier network, referrals to the third-level unit are made by the second-level regional autism centers, which coordinate and, where required, escalate cases identified by the first-level outpatient and community services.

To optimize the planning of the intensive intervention, a standardized admission and assessment protocol was developed. The process, summarized below in its main phases, includes:

- Receipt of the admission request from the referring autism centers and neuropsychiatric services;
- Initial contact with the referring clinicians for a preliminary description of the case;
- Interviews with family members, caregivers, and/or professionals already involved in the patient’s care, together with the administration of a pre-admission information questionnaire aimed at collecting data on adaptive functioning, educational profile, academic abilities, and characteristics of the challenging behaviors;
- Preliminary visit to the unit by family members and, whenever possible, by the patient, during which information regarding the stay and therapeutic program is provided;
- Admission to the unit with reception by the case manager (psychologist), the reference educator, and the nursing coordinator, including support for environmental adaptation and logistical organization;
- During the first week, completion of routine clinical examinations (performed by the unit’s medical staff, i.e., psychiatrists and, where indicated, other on-site specialists) and initiation of systematic observations (initiated by the reference educators and nursing staff, in coordination with the behavior analyst) concerning autonomy, existing abilities, and behavioral criticalities;
- After the first week, implementation of structured observation sessions with the behavior analyst and the reference educator;
- Development of the first educational and behavioral recommendations, together with the definition of data collection procedures;
- Every 15 days, multidisciplinary micro-team meetings aimed at reviewing collected data, evaluating intervention effectiveness, and redefining clinical and educational goals;
- Ongoing collaboration with an external supervising psychologist;
- Continuous coordination with the territorial services responsible for the patient’s care, to share treatment progress and therapeutic goals.

The psychoeducational approach adopted by the unit is based on the principles of ABA, integrated within an intensive and individualized model aimed at reducing dysfunctional behaviors and enhancing adaptive skills. Within the inpatient setting, this approach is intended to provide an intensive intervention delivered within the expected duration of stay, while also preparing the patient for discharge and future placement in residential and/or home settings. The main phases are described in **Table 1**.

**Table 1:** Summary of the unit’s operational procedure.

Phase	Main activities	Included assessments
Admission referral	Referral from adult or child autism centers or child neuropsychiatry services	–
Initial consultation with referring clinicians	Collection of preliminary clinical information	–
Family/caregiver interview	Clinical interview and pre-admission questionnaire	Preliminary observational assessment
Preliminary visit to the unit	Information regarding inpatient stay and treatment program	–
Admission to the unit	Initial assessment and environmental adaptation	–
First week of admission	Clinical examinations and systematic observations	Cognitive assessment and observational scales
Structured observation sessions	Behavioral observations conducted by the behavior analyst	Functional behavioral assessment
Development of behavioral and educational recommendations	Definition of data collection procedures	Observational data analysis
Multidisciplinary team meetings	Data review and revision of clinical goals	Adaptive and behavioral monitoring
Coordination with community services	Sharing treatment progress and care planning	–
Pre-discharge assessment	Preparation for post-discharge transition	Reassessment of adaptive functioning and cognitive profile

### Psychoeducational intervention

The psychoeducational intervention adopted by the unit was based on the principles of ABA (19, 20), which includes:

- Functional behavior assessment aimed at identifying antecedent and consequent variables maintaining a behavior (18);
- Breakdown of complex skills into smaller and teachable units through task analysis and prompting procedures;
- Systematic use of reinforcement to increase the frequency of desired behaviors, in accordance with the principles of operant conditioning;
- Procedures aimed at reducing challenging behaviors through differential interventions, extinction, or manipulation of environmental contingencies;
- Continuous data monitoring, including graphical evaluations and trend analyses to guide clinical decision-making (20);
- Individualization of the intervention program, adapted to the characteristics of the individual and modified according to observed progress.

The methodological approach adopted by the unit is aligned with the guidelines of Società Italiana di Neuropsichiatria dell’Infanzia e dell’Adolescenza (2005) and the Guidelines of the Italian National Institute of Health (Istituto Superiore di Sanità, 2025), which identify behavioral interventions as the treatment of choice for autism, highlighting in particular the evidence supporting ABA in improving intellectual functioning, language abilities, and adaptive behaviors.

### Outcome measures and diagnostic characterization

Admission and discharge assessments were conducted by the Adult Autism Outpatient Clinic of the ASL Città di Torino, the regional coordinating center, before admission (T0) and at discharge (T1). The T0 and T1 outcome assessments were completed with the support of a healthcare professional or caregiver who had extensive knowledge of the participant’s daily functioning, thereby providing collateral information on the participant’s clinical and functional status at the time of assessment. All participants were already receiving care from the Adult Autism Outpatient Clinic of the ASL Città di Torino and had previously undergone the standard diagnostic pathway adopted by the service (12). The core diagnostic pathway included cognitive assessment using the Wechsler Adult Intelligence Scale–Fourth Edition (WAIS-IV) (45), autism-specific diagnostic assessment using the Autism Diagnostic Observation Schedule, Second Edition (ADOS-2) (46), and the Autism Diagnostic Interview–Revised (ADI-R) (47). When clinically indicated, additional structured assessments were administered to support differential diagnosis and characterize co-occurring conditions. In participants presenting with symptoms of inattention or hyperactivity, the Diagnostic Interview for ADHD in Adults, Fifth Edition (DIVA-5) (48), was used to assess ADHD symptoms during both childhood and adulthood, with collateral information from a caregiver when available. The Structured Clinical Interview for DSM-5 Personality Disorders (SCID-5) (49) was also administered when further assessment of personality functioning was required to distinguish autism-related features from symptoms attributable to a co-occurring personality disorder. The outcome measures were completed by trained clinicians from the multidisciplinary team of the Adult Autism Outpatient Clinic.

When challenging behaviors escalated to a level requiring admission to the tertiary-level service, participants underwent the T0 assessment protocol described below. Co-occurring psychiatric and medical conditions were recorded by the clinician responsible for completing the admission protocol, based on diagnoses and other information documented in the available clinical records.

The outcome battery comprised:

- Brief Psychiatric Rating Scale (BPRS) (50): assessing the severity of 24 different psychiatric symptoms using a 7-point Likert scale.
- Aberrant Behavior Checklist (ABC) (51): a 58-item informant-rated scale comprising five subscales, namely irritability, lethargy/social withdrawal, stereotypic behavior, hyperactivity/noncompliance, and inappropriate speech.
- Clinical Global Impression (CGI) (52): evaluating global functioning through a 7-point severity scale. It also includes a 7-point score assessing change following treatment, ranging from worsening of the clinical condition to marked improvement.
- Social and Affective Functional Support Scale (SSAF) (53): collecting information regarding challenging behaviors, including their intensity and frequency, social influences on behavior, and reinforcement variables.

### Data analysis

Statistical analyses were conducted to evaluate the impact of the intervention by comparing baseline (T0) and discharge (T1) assessments. Given the within-subject design, differences between the two assessment time points were analyzed using two-tailed paired-samples t-tests. Analyses were conducted separately for each subscale of the instruments used for patient assessment. Descriptive statistics were calculated using all valid observations available at each assessment time point, whereas each paired-samples t-test included only participants with valid scores at both T0 and T1 for the corresponding outcome. All statistical analyses were performed using IBM SPSS Statistics 30 (https://www.ibm.com/support/pages/downloading-ibm-spss-statistics-30). The significance threshold was set at α = .05; t values, degrees of freedom (df), significance levels, and effect sizes (Cohen’s dz) were reported, and results outside the α = .05 threshold were interpreted descriptively with reference to their effect sizes, rather than being described as statistical trends. Given the number of subscale comparisons performed relative to the sample size, no correction for multiple comparisons was applied, and the analyses are therefore explicitly exploratory rather than confirmatory.

## Results

The study sample consisted of the first 10 patients with ASD who were consecutively admitted following implementation of the program and who completed it. All participants were adult males, aged 20.2-43.0 years (M = 27.8 years, SD = 9.5). No sex-based eligibility criterion was applied; the exclusively male composition reflected the sex distribution of patients referred to and admitted to the service during the study period. Clinical and sociodemographic characteristics, together with the duration of hospitalization and comorbidities, are reported in **Table 2**.

**Table 2:** Clinical and sociodemographic characteristics of the study participants.

Subject ID	Sex	Age (T0)	Severity level	FSIQ	Comorbidities	Medications	Length of hospital stay (days)
1	M	43.0	3	46	No	No	104
2	M	21.1	3	66	No	No	111
3	M	21.3	2	46	No	No	119
4	M	21.9	2	43	No	No	113
5	M	41.8	3	Not assessable	Pica	Trazodone; sodium valproate; risperidone; melatonin	110
6	M	20.2	2	46	No	Risperidone; sodium valproate; clonazepam; clotiapine; quetiapine; chlorpromazine; hydroxyzine	114
7	M	22.0	3	<40	ADHD; developmental and epileptic encephalopathy of genetic etiology; Pica	Sodium valproate; olanzapine; lorazepam; flurazepam	124
8	M	38.9	3	<40	No	No	57
9	M	26.6	3	<40	Language disorder	Aripiprazole; delorazepam; levomepromazine; trazodone; zolpidem	186
10	M	21.2	1	80	Borderline personality disorder	No	108

ADHD, attention-deficit/hyperactivity disorder; FSIQ, Full-Scale Intelligence Quotient; T0, pre-admission assessment.

Descriptive statistics were calculated for all clinical measures at baseline (T0; pre-intervention) and discharge (T1; post-intervention). The number of valid observations varied across instruments, subscales, and assessment time points because of missing data. Descriptive statistics were therefore calculated separately for each measure using all available observations at the corresponding assessment time point. **Table 3** summarizes the available sample size, mean, and standard deviation for the principal dimensions of the BPRS, ABC, CGI, and SSAF at T0 and T1. For the inferential analyses, each paired-samples t-test included only participants with valid scores at both assessment time points for the corresponding outcome. Accordingly, the findings should be interpreted cautiously given the small and variable number of complete pairs and the exploratory nature of the analyses.

**Table 3:** Descriptive statistics for clinical and behavioral measures assessed at baseline (T0; pre-intervention) and discharge (T1; post-intervention).

Instrument	Variable/subscale	N (T0)	M (T0)	SD (T0)	N (T1)	M (T1)	SD (T1)
BPRS	Somatic concern	9	3.11	1.96	8	1.88	2.10
BPRS	Anxiety	9	5.22	1.99	9	3.33	1.73
BPRS	Motor tension	10	5.30	1.25	10	3.00	1.94
BPRS	Distractibility	8	5.50	1.20	9	3.22	2.17
BPRS	Bizarre behavior	9	5.67	1.73	10	3.10	2.13
BPRS	Emotional withdrawal	10	4.90	1.85	10	3.00	1.05
BPRS	Conceptual disorganization	8	4.63	1.69	9	3.44	1.67
ABC	Irritability (ABC-I)	9	17.56	7.00	10	13.20	10.36
ABC	Lethargy/Social withdrawal (ABC-II)	10	20.80	9.90	10	11.60	9.16
ABC	Stereotypic behavior (ABC-III)	10	8.00	4.19	10	5.00	6.50
ABC	Hyperactivity (ABC-IV)	10	20.90	10.17	10	11.40	10.78
ABC	Inappropriate speech (ABC-V)	10	6.90	8.50	10	1.90	2.51
CGI	Severity	8	6.00	0.53	10	5.20	0.79
SSAF	Social support (no. of “yes” responses)	10	13.20	6.12	8	11.00	5.55

BPRS, Brief Psychiatric Rating Scale; ABC, Aberrant Behavior Checklist; CGI, Clinical Global Impression; SSAF, Social and Affective Functional Support Scale; M, mean; SD, standard deviation; T0, pre-intervention; T1, post-intervention.

### Brief psychiatric rating scale

Descriptive statistics from the BPRS indicated that the highest mean baseline scores, relative to the other subscales, were observed for bizarre behavior (M = 5.67, SD = 1.73, N = 9), distractibility (M = 5.50, SD = 1.20, N = 8), motor tension (M = 5.30, SD = 1.25, N = 10), anxiety (M = 5.22, SD = 1.99, N = 9), self-neglect (M = 5.17, SD = 1.94, N = 6), mannerisms and posturing (M = 5.10, SD = 1.60, N = 10), and emotional withdrawal (M = 4.90, SD = 1.85, N = 10).

At discharge, mean scores were lower than at baseline across most BPRS domains. Anxiety decreased to M = 3.33 (SD = 1.73, N = 9), motor tension to M = 3.00 (SD = 1.94, N = 10), distractibility to M = 3.22 (SD = 2.17, N = 9), bizarre behavior to M = 3.10 (SD = 2.13, N = 10), emotional withdrawal to M = 3.00 (SD = 1.05, N = 10), and motor hyperactivity to M = 2.50 (SD = 1.78, N = 10). Reductions were also observed in unusual thought content, from M = 3.86 (SD = 2.27, N = 7) to M = 2.38 (SD = 2.00, N = 8), and conceptual disorganization, from M = 4.63 (SD = 1.69, N = 8) to M = 3.44 (SD = 1.67, N = 9).

**Figure 1:**
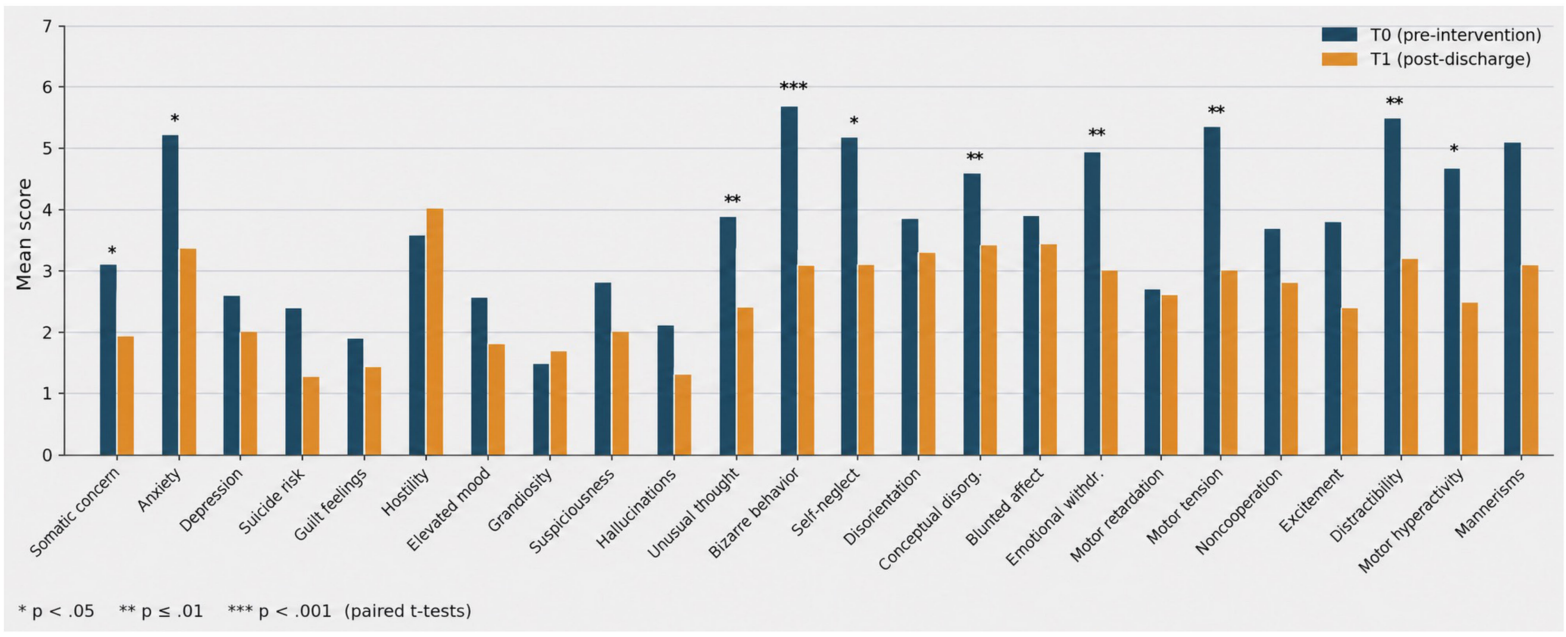
Mean brief psychiatric rating (BPRS) subscale scores before (T0) and after (T1) the intervention. Asterisks denote uncorrected paired-samples t-tests (*p <.05; **p ≤.01; ***p <.001). disorg, disorganization; withd, withdrawal.

Paired-samples t-test analyses of the BPRS subscales revealed statistically significant reductions in several symptom domains (**Table 4**). Significant improvements were observed for anxiety, t(7) = 3.15, p = .016, dz = 1.11, and somatic concern, t(6) = 2.59, p = .041, dz = 0.98. At the cognitive level, significant reductions were observed for unusual thought content, t(6) = 3.74, p = .010, dz = 1.41, and conceptual disorganization, t(6) = 4.21, p = .006, dz = 1.59 (**Figure 1**).

**Table 4:** Results of paired-sample t-tests comparing T0 (pre-intervention) and T1 (post-intervention) scores on the Brief Psychiatric Rating (BPRS) subscales. .

Variable	N (paired)	t(df)	p	dz
Statistically significant findings				
Somatic concern	7	2.59 (6)	.041	0.98
Anxiety	8	3.15 (7)	.016	1.11
Unusual thought content	7	3.74 (6)	.010	1.41
Bizarre behavior	9	5.12 (8)	<.001	1.71
Self-neglect	6	3.22 (5)	.023	1.32
Conceptual disorganization	7	4.21 (6)	.006	1.59
Emotional withdrawal	10	3.61 (9)	.006	1.14
Motor tension	10	4.27 (9)	.002	1.35
Distractibility	7	5.30 (6)	.002	2.00
Motor hyperactivity	9	2.83 (8)	.022	0.94
Non-significant findings				
Mannerisms and posturing	10	2.24 (9)	.052	0.71
Excitement	10	2.09 (9)	.066	0.66
Suicide risk	4	2.32 (3)	.103	1.16
Hallucinations	7	1.62 (6)	.156	0.61
Non-cooperation	10	1.45 (9)	.182	0.46
Guilt feelings	6	1.39 (5)	.224	0.57
Suspiciousness	9	1.22 (8)	.256	0.41
Elevated mood	9	1.11 (8)	.301	0.37
Blunted affect	8	1.11 (7)	.303	0.39
Depression	9	0.83 (8)	.430	0.28
Disorientation	10	0.67 (9)	.521	0.21
Hostility	10	−0.46 (9)	.657	0.15
Grandiosity	9	−0.22 (8)	.834	0.07
Motor retardation	10	0.13 (9)	.899	0.04

df, degrees of freedom; dz, Cohen’s d for paired samples; N (paired), number of participants with valid scores at both T0 and T1. Each analysis included only participants with valid scores at both T0 and T1 for the corresponding subscale. Positive t values indicate lower scores at T1 than at T0; negative t values indicate higher scores at T1. Cohen’s dz is reported as an absolute magnitude. No correction for multiple comparisons was applied.

Behavioral dimensions also showed statistically significant reductions. These included bizarre behavior, t(8) = 5.12, p <.001, dz = 1.71, self-neglect, t(5) = 3.22, p = .023, dz = 1.32, distractibility, t(6) = 5.30, p = .002, dz = 2.00, and motor hyperactivity, t(8) = 2.83, p = .022, dz = 0.94. Significant reductions were also observed for motor tension, t(9) = 4.27, p = .002, dz = 1.35, and emotional withdrawal, t(9) = 3.61, p = .006, dz = 1.14.

No statistically significant differences were observed for depression, guilt, hostility, elevated mood, grandiosity, suspiciousness, hallucinations, disorientation, blunted affect, motor retardation, or non-cooperativeness (all p >.05). Mannerisms and posturing did not reach the statistical significance threshold, t(9) = 2.24, p = .052, dz = 0.71, although the effect size indicated a moderate-to-large within-subject reduction. Excitement also showed a non-significant reduction with a moderate effect size, t(9) = 2.09, p = .066, dz = 0.66. Suicide risk showed a large but non-significant reduction, t(3) = 2.32, p = .103, dz = 1.16; this estimate was based on only four complete pairs and should therefore be interpreted cautiously.

### Aberrant behavior checklist

Descriptive statistics from the ABC indicated the following baseline mean scores: Irritability (ABC-I), M = 17.56 (SD = 7.00, N = 9); Lethargy/Social Withdrawal (ABC-II), M = 20.80 (SD = 9.90, N = 10); Stereotypic Behavior (ABC-III), M = 8.00 (SD = 4.19, N = 10); Hyperactivity/Noncompliance (ABC-IV), M = 20.90 (SD = 10.17, N = 10); and Inappropriate Speech (ABC-V), M = 6.90 (SD = 8.50, N = 10). Because the ABC subscales contain different numbers of items, their raw scores were not interpreted as directly comparable indicators of the relative severity of impairment across domains. At discharge, a reduction in the mean score was observed for every ABC subscale. Irritability/Agitation/Crying decreased to M = 13.20 (SD = 10.36, N = 10), Lethargy/Social Withdrawal to M = 11.60 (SD = 9.16, N = 10), and Hyperactivity/Noncompliance to M = 11.40 (SD = 10.78, N = 10). Stereotypic Behavior decreased to M = 5.00 (SD = 6.50, N = 10), while Inappropriate Speech decreased to M = 1.90 (SD = 2.51, N = 10).

**Figure 2:**
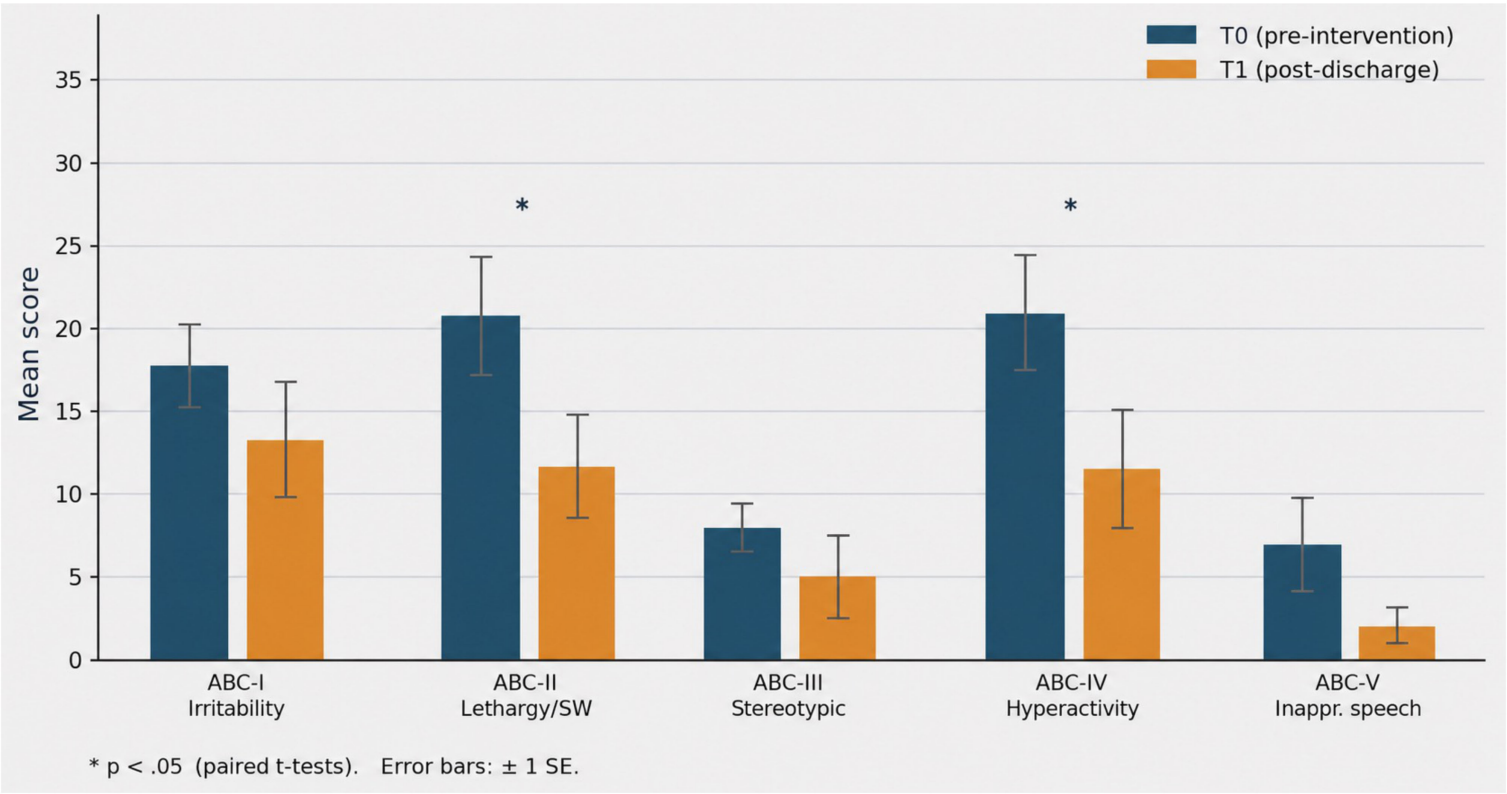
Mean aberrant behavior checklist (ABC) subscale scores before (T0) and after (T1) the intervention; error bars denote ±1 standard error (SE). Asterisks denote uncorrected paired-samples t-tests (*p <.05). ABC, Aberrant Behavior Checklist; ABC-I, Irritability; ABC-II, Lethargy/Social Withdrawal; ABC-III, Stereotypic Behavior; ABC-IV, Hyperactivity; ABC-V, Inappropriate Speech; SE, standard error; SW, social withdrawal.

Paired-samples t-test analyses revealed statistically significant reductions in Lethargy/Social Withdrawal (ABC-II), t(9) = 2.74, p = .023, dz = 0.87, and Hyperactivity/Noncompliance (ABC-IV), t(9) = 2.39, p = .041, dz = 0.76 (**Figure 2**). These findings indicated large within-subject reductions in social withdrawal and hyperactivity/noncompliance.

The reduction in Irritability (ABC-I) was not statistically significant, t(8) = 1.10, p = .302, dz = 0.37. Similarly, no statistically significant differences were observed for Stereotypic Behavior (ABC-III), t(9) = 1.28, p = .234, dz = 0.40, or Inappropriate Speech (ABC-V), t(9) = 1.78, p = .109, dz = 0.56. The corresponding effect sizes indicated small-to-moderate reductions in Irritability/Agitation/Crying and Stereotypic Behavior and a moderate reduction in Inappropriate Speech.

### Clinical global impression scale

Descriptive statistics from the Clinical Global Impression–Severity scale (CGI-S) indicated a baseline mean severity score of M = 6.00 (SD = 0.53, N = 8), corresponding approximately to a “severely ill” clinical rating. At discharge, the mean CGI-S score was M = 5.20 (SD = 0.79, N = 10). The Clinical Global Impression–Improvement scale (CGI-I), assessed exclusively at T1, showed a mean score of M = 2.56 (SD = 1.67, N = 9), corresponding on average to a clinical judgment between “much improved” and “minimally improved”.

The paired-samples analysis of CGI-S did not demonstrate a statistically significant reduction in overall clinical severity between T0 and T1, t(7) = 1.82, p = .111, dz = 0.64. Nevertheless, the descriptive reduction and corresponding effect size indicated a moderate within-subject change.

### Perceived social support scale

For Part IV of the SSAF, which assesses social influences on behavior, the baseline mean number of affirmative responses was M = 13.20 (SD = 6.12, N = 10). At discharge, the mean was M = 11.00 (SD = 5.55, N = 8).

The paired-samples analysis did not reveal a statistically significant difference between T0 and T1, t(7) = 1.01, p = .348, dz = 0.36, indicating a small-to-moderate reduction in the number of affirmative responses.

## Discussion

The present study aimed to evaluate the preliminary effectiveness of a clinical intervention delivered in the residential unit for severe behavioral disorders in autistic individuals, using a pre-post inpatient design and examining a broad spectrum of psychopathological, behavioral, and psychosocial functioning indicators. Rather than a uniform improvement, the findings indicated a differentiated pattern of change. Specifically, the post-intervention change was concentrated across a coherent set of behavioral-disorganization and agitation dimensions (i.e., motor tension, distractibility, bizarre behavior, conceptual disorganization, and anxiety), paralleled by reductions in emotional/social withdrawal and hyperactivity. By contrast, the more trait-like, affective, and global dimensions remained essentially unchanged.

The dimensions that improved are precisely those most plausibly operant in nature; that is, evoked and maintained by identifiable environmental contingencies and antecedents (14, 16); therefore, the ones most directly addressable by the methods used in the unit. Functional behavioral assessment identifies the variables that maintain a given challenging behavior, after which antecedent modification, extinction of the maintaining contingency, and differential reinforcement of an equally efficient, socially appropriate alternative can shift the behavioral repertoire away from the challenging topography (54–56). When that alternative is communicative, this is the logic of functional communication training, one of the most robust function-based treatments for severe behavior problems (57–59). Importantly, this rationale is also supported, although by a smaller literature, in autistic adults and in adults with intellectual and developmental disabilities (60, 61). On this account, the reductions in agitation, motor tension, and disorganized behavior are best read not as the direct “treatment” of discrete symptoms but as a reorganization of the behavioral repertoire toward functionally equivalent, better-regulated responses.

However, this interpretation must be held with caution, because a competing and non-exclusive explanation may operate in parallel. A highly structured, predictable, low-arousal inpatient environment removes or attenuates many of the antecedents and establishing operations that ordinarily evoke challenging behavior (62, 63). Part of the improvement observed at discharge may therefore reflect environmentally supported behavior under tight stimulus control rather than newly acquired, portable self-regulation (64). A single discharge assessment cannot distinguish durable, generalized learning from context-dependent suppression that may attenuate once the person returns to a less structured setting (65, 66). This is the classical problem of generalization and maintenance in behavior analysis (65, 67): gains do not transfer automatically and must be actively programmed across settings and caregivers (59, 66, 68). So framed, the post-discharge continuity built into the present model is not an administrative detail but a theoretically necessary condition for the observed gains to persist (63). This consideration extends beyond autism-specific behavior. Three of the dimensions showing significant change (i.e., motor tension, distractibility, and motor hyperactivity) together with the ABC Hyperactivity/Noncompliance subscale, describe motor restlessness, attentional dysregulation, and overactivity, and therefore overlap at the symptom level with the phenomenology of ADHD, which co-occurs in a substantial proportion of autistic individuals and is difficult to distinguish from autism-related presentations in adults with intellectual disability and limited spoken language (69, 70). Because the instruments employed here index the topography of observable behavior rather than its etiology, and because environmental structuring is itself a core component of the non-pharmacological management of attentional and hyperactive presentations, the admission may have acted in part on broader transdiagnostic dimensions of attentional, arousal, and behavioral dysregulation rather than exclusively on behavior specifically linked to autism. Two features of the present pattern, however, argue against comorbid ADHD as its principal explanation. First, the significant changes extended well beyond the dimensions with an ADHD counterpart, encompassing bizarre behavior, conceptual disorganization, unusual thought content, self-neglect, emotional withdrawal, and anxiety; these included the second, third, and fourth largest effect sizes observed. Second, a co-occurring diagnosis of ADHD was documented in only one of the ten participants. A comparable consideration applies to personality-related features: hostility and non-cooperation, the dimensions corresponding most closely to them, showed no significant change. At the same time, no ADHD-specific instrument was administered at T0 and T1, so change in attentional symptoms was not measured directly, and attentional and impulse-regulation difficulties in this population are liable to diagnostic overshadowing, so under-recognition cannot be excluded. Transdiagnostic dysregulation may therefore have contributed to, without fully accounting for, the improvement observed, a qualification that concerns what the change should be attributed to rather than whether it occurred. A further consideration concerns attribution. The admission is intrinsically multimodal, consistent with multidisciplinary approaches recommended for complex, challenging behaviors in neurodevelopmental disorders (71). Specifically, the behavioral program is delivered alongside systematic medical and pharmacological re-evaluation and a comprehensive environmental restructuring (71, 72). Acute behavioral deterioration in autistic adults is frequently precipitated or sustained by unrecognized somatic or psychiatric conditions, and addressing these is itself associated with clinical improvement (13, 73, 74). The present design cannot isolate the contribution of the behavioral component from these co-occurring elements. Therefore, the results are most accurately described as the effect of an integrated program, not of ABA in isolation.

Conversely, more stable or structural dimensions, such as depression, blunted affect, or grandiosity, did not show significant changes, suggesting lower short-term sensitivity to change. Several factors plausibly converge here. These dimensions may tap comparatively trait-like features and internalizing or psychotic-spectrum states that are less directly contingent on immediate environmental events and often show greater temporal complexity in the autistic population (75, 76). They are also more difficult to measure reliably in this population, where reduced introspective report and emotion-identification difficulties may affect the assessment of affective states (77–79), and where the risk of diagnostic overshadowing (i.e., the tendency to attribute affective or psychopathological signs to autism or intellectual disability itself) can blunt the detection of genuine change (80). This finding is consistent with the possibility that the behavioral components of the integrated program preferentially affected observable domains, in alignment with the wider ABA literature (14, 16, 19), whereas internalizing and affective symptoms, often characterized by greater stability and clinical complexity, may require different therapeutic approaches, such as adapted psychological interventions, pharmacological treatment of co-occurring psychiatric symptoms, or longer intervention periods to demonstrate significant improvement (81–83).

The ABC findings converge with those of the BPRS. Because the two instruments differ in source and item content yet point to the same selective signal, this convergence increases confidence that the effect is not an artifact of a single scale, while leaving the mechanistic account above unchanged. The reductions in social withdrawal and hyperactivity are consistent with the description of these manifestations as distinct topographies of a common underlying class of challenging behaviors (11). Although the Irritability subscale showed a moderate reduction, this change did not reach statistical significance. Inappropriate speech did not demonstrate significant changes, suggesting greater resistance to treatment in more structured communicative domains. A useful distinction could clarify this pattern. The communicative function of a challenging behavior may be directly targetable through functional communication training and has contributed to the behavioral reductions described above (59). By contrast, formal expressive language and pragmatic communication skills constitute a core and highly consolidated feature of autism in adulthood and change only slowly (5, 84, 85). Previous literature indicates that interventions targeting communication skills during the developmental age are associated with more favorable adult functional outcomes. Conversely, intervention on these abilities in adulthood may be more complex and require longer learning periods, as communicative patterns tend to be more consolidated and generalized across life contexts (5, 84, 86, 87). The absence of change in inappropriate speech is coherent with this view: a short admission can alter the function that maintains a behavior more readily than it can reshape entrenched communicative form.

Global assessment through the CGI did not reveal a statistically significant reduction in overall clinical severity, despite a trend toward improvement and a moderate effect size. This may be interpreted in light of the nature of the CGI, namely a broad, clinician-rated global impression scale that synthesizes overall illness severity or improvement into a small number of global ratings and is commonly used alongside more domain-specific symptom scales (88–90). As such, it may be less sensitive to circumscribed behavioral change than multi-item instruments targeting specific symptoms or behavioral domains. The severity of autism and the associated global functional impairment, primarily related to the autistic condition rather than to psychopathological or behavioral symptoms alone, are unlikely to change significantly within only a few months; longitudinal work indicates substantial continuity in autism-related features and support needs from childhood into adolescence and adulthood, even though specific behaviors and adaptive outcomes may vary across time (5, 84, 85, 91). The individual’s underlying autism severity and general support needs may thus remain stable while the challenging behaviors improve. At the same time, it should be noted that this comparison rested on a markedly small analyzable subsample, so the analysis was likely underpowered to detect an effect of this magnitude; therefore, the lack of statistical significance could reflect limited power at least as much as a genuine absence of clinical change. Similarly, no statistically significant changes emerged regarding perceived social support, assessed through the SSAF, consistent with the fact that perceived social support reflects a subjective appraisal of available relational support and would not necessarily be expected to shift over a brief inpatient admission unless the person’s social network or post-discharge support ecology also changes (92, 93). Also in this case, the paired analysis was based on only a limited number of observations, and this small subsample size further limits the power to detect possible change, independent of any substantive interpretation.

The temporary inpatient management of autistic individuals with challenging behaviors at IRCCS Maugeri, embedded within a coordinated regional territorial network, represents an innovative intervention model that, according to these preliminary clinical findings, appears to yield promising outcomes and supports the development of structured and specialized care pathways for these severe clinical conditions. Unlike other facilities currently operating at the national level, this model includes both upstream intervention through territorial healthcare teams and downstream intervention following discharge, involving gradual integration into post-discharge projects connected. This creates a coordinated “Piedmont Model” characterized by an integrated care pathway and an extensive outpatient territorial network coordinated by the two regional autism centers, interfacing directly with IRCSS Maugeri. As argued above, this upstream-downstream architecture is also the feature most likely to determine whether inpatient gains endure, since it provides the cross-setting continuity that the generalization of behavioral change requires.

Two years of operation have also yielded several practical observations about implementing such a model. During admission, staff members receive both external clinical supervision and internal psychological supervision focused on the emotional well-being of the multidisciplinary team, two fundamental elements in severe disability care settings, whose absence may contribute to maladaptive behavioral responses among healthcare workers. Before employment, staff members undergo a one-week “subjective trial” period, allowing them to assess their emotional suitability for this type of work, and the unit maintains a highly structured organization of staff responsibilities, which has progressively reduced staff turnover over time. The unit also collaborates with external organizations, promoting occupational activities within the facility’s garden areas. Throughout the pathway, an internal case manager for each patient (supported by a psychologist, behavior analyst, and psychiatrist) is considered essential, and the availability on site of other medical specialties (physiatry, cardiology, neurology), together with the nearby general hospital in Novara, allows direct evaluation of organic contributors to behavioral crises.

Overall, the findings delineate a coherent pattern: the intervention appears to be associated with improvement in the most dynamic and modifiable dimensions, whereas more global or structural dimensions appear less sensitive to short-term change. Nevertheless, they must be interpreted in light of several methodological limitations. First, the small sample size limits both generalizability and statistical power. Second, the absence of a control group precludes definitive causal attribution, as time-related or uncontrolled factors cannot be excluded. Third, the analyses were restricted to participants who completed the hospitalization program and underwent both baseline and discharge assessments. Consequently, the findings may not generalize to patients who discontinue the program. Some outcome-level data were also unavailable, resulting in variable numbers of complete T0-T1 pairs across measures and reducing the precision and statistical power of individual analyses. Fourth, a large number of subscale comparisons were performed without correction for multiple testing; consequently, some of the nominally significant results reported here may constitute false positives. Given this, the exploratory, hypothesis-generating nature of these analyses should be underscored, and interpretive weight should be placed on the overall consistency of the pattern of effect sizes across related dimensions and instruments, rather than on the statistical significance of any single comparison. Fifth, the exclusively male composition of the sample limits the generalizability of the findings to autistic women. This limitation is particularly relevant given evidence that females meeting criteria for ASD are less likely to receive a clinical diagnosis and may follow different diagnostic and referral pathways, partly because of differences in clinical presentation and camouflaging (94, 95). The present study cannot determine whether the absence of female participants reflects differences in the prevalence or severity of challenging behaviors, referral and admission practices, access to highly specialized services, or other contextual factors. Future multicenter studies should therefore actively examine the accessibility, clinical presentation, and treatment outcomes of autistic women with severe challenging behaviors. Sixth, feasibility and acceptability were not evaluated using standardized or prospectively defined measures. Similarly, the study did not include a dedicated systematic procedure for monitoring adverse events or unintended effects. Finally, and most importantly for interpretation, the absence of any assessment beyond discharge leaves the durability and cross-setting generalization of the gains untested, and the multimodal nature of the admission prevents isolation of the behavioral component from its medical and environmental co-interventions. Despite these limitations, the findings provide valuable preliminary evidence regarding the intervention, particularly in selected behavioral and disorganization-related domains. Future research should pre-specify a small set of primary outcomes, incorporate a control or comparison group and, where feasible, blinded outcome assessment, recruit larger and sex-diverse samples across multiple sites, and add post-discharge follow-up, along with adaptive functioning, quality of life, and caregiver burden measures. Prospective studies should also systematically document treatment completion, reasons for discontinuation, and any adverse or unintended effects. Such designs would allow the field to move from documenting that change occurs during admission to establishing whether it persists and generalizes afterwards, and to begin disentangling which components of the integrated program drive it.

## Conclusions

This study provides preliminary, exploratory evidence that a specialized, time-limited inpatient program for severe challenging behaviors in autistic adults, delivered within an integrated three-tier regional network, is associated with measurable clinical improvement. Importantly, that improvement was selective rather than global; it concentrated in observable, operant dimensions of behavioral disorganization and agitation. Because the inpatient pathway combined behavioral intervention with medical and pharmacological re-evaluation and environmental restructuring, the observed changes are best understood as reflecting the integrated program as a whole, rather than the isolated effect of its ABA-based component. Moreover, as no post-discharge assessment was conducted, improvement refers exclusively to changes observed during hospitalization. Whether these gains persist over time, generalize to everyday settings, or depend on the highly structured inpatient environment remains to be established. Therefore, these findings should be read as hypothesis-generating. The uncontrolled design, the modest and exclusively male sample, and the absence of post-discharge measurement preclude causal claims and leave the durability and generalization of the gains untested. They nonetheless indicate that dedicated, coordinated care pathways are a promising direction for a markedly underserved population. The model’s distinctive upstream-downstream architecture, linking territorial services before admission with structured continuity after discharge, is precisely the feature most likely to determine whether inpatient gains endure.

## Data Availability

The raw data supporting the conclusions of this article will be made available by the authors, without undue reservation.
